# Multiple sources of bias confound functional enrichment analysis of global -omics data

**DOI:** 10.1186/s13059-015-0761-7

**Published:** 2015-09-07

**Authors:** James A. Timmons, Krzysztof J. Szkop, Iain J. Gallagher

**Affiliations:** Division of Genetics and Molecular Medicine, King’s College London, Guy’s Hospital, Great Maze Pond, London, SE1 9RT UK; School of Natural Sciences, University of Stirling, Stirling, FK9 4LA UK

## Abstract

Serious and underappreciated sources of bias mean that extreme caution should be applied when using or interpreting functional enrichment analysis to validate findings from global RNA- or protein-expression analyses.

Large gene-expression datasets are used for various purposes, including the investigation of fundamental rules that govern transcription, the prediction of responses to drug treatment and studies of physiological adaptation. A common strategy for analyzing such gene-expression data is the identification of differentially regulated genes using a statistical comparison of samples from different conditions.

Differential expression analysis produces a list of genes (often several hundred to several thousand) that meet some criteria for statistical significance. These criteria should ideally balance the false-positive and false-negative rates, but their choice is typically ad hoc, especially in studies of limited sample size and/or size effect. It is important to place such a gene list of differentially expressed genes into an interpretable structure, which hopefully reflects the underlying biological processes that are uniquely regulated in the experiment [1]. Clinical transcriptomics studies in particular regularly use the process of ‘grouping’ genes into functional categories, combined with a statistical test, to support the claim that an analysis is robust [2, 3].

Most frequently, the functional categories are identified using classification schemes such as that of the Gene Ontology (GO) consortium [4] (MSigDB, GeneSigDB and Ingenuity Pathway Analysis (IPA) are other examples). Discovery that the list of regulated genes contains an overrepresentation for one or more biological ‘functions’ can identify the underlying mechanism for the difference between conditions, giving the data analysis a ‘plausibility factor’. This evidence is coupled with a statistical value that summarizes the likelihood that the overrepresentation did not occur by chance. Functional enrichment analysis is, therefore, a widely used strategy to legitimize -omic studies and, consequently, impacts the peer review process as a result of reviewer perceptions and the statistical acceptability of the analysis [2, 3].

Two studies published in the last five years identified significant sampling bias in functional enrichment analysis [5, 6]. Specifically, both indicated that the ‘discovery’ of significant and plausible functional enrichment profiles could be achieved in almost every analysis, regardless of how the regulated gene list was selected. However, the discovery of ‘sampling bias’ has had little impact on how -omic data are interpreted or reported.

To understand the nature of this ‘sampling bias’ problem, first appreciate that the statistical identification of a functional category (for example, ‘muscle development’) from the list of differentially regulated genes implies that more of the genes on the list belong to that category than one would expect from a random sample of ‘all genes’. Critically, the statistical test for the enrichment of a particular functional category utilizes a reference gene list, and a problem arises if the content of this list does not adjust for sampling bias.

Sampling bias (including reflecting features of the DAVID (Database for Annotation, Visualization, and Integrated Discovery) annotation status) can arise in three different ways. First, every RNA detection technology, including RNA sequencing, has a biased representation of the gene ontology structure. For example, the Affymetrix Human Genome U133 Plus 2.0 GeneChip has proportionately more genes linked to ‘acetylation’ (*P* < 7 × 10^−51^) than does the genome (as defined by the DAVID online gene annotation tool [7]), whereas the Agilent 44 K chip has proportionately more genes linked to ‘mutagenesis site’ (*P* <2 × 10^−46^). In fact, hundreds of gene categories are massively ‘enriched’ in functional classifications on every microarray—this is called technology bias.

Furthermore, not all genes can be detected with equal reliability, to the extent that some genes are never detected as being ‘regulated’ (the signal never changes). This is detection bias, which can reflect aspects of the transcriptomics technology or the sequence of the transcript that is being probed.

There is also a third and more obvious bias. The transcriptome of a given cell type or tissue is highly specialized, to the point that it can be used to determine the identity of an unknown RNA profile efficiently; this is referred to as biological bias.

We use two published examples to illustrate a very common problem. The first is a study of transcriptional responses to disuse in human skeletal muscle [3]. Here, the reported ontologies reflect the strong biological bias that muscle has for oxidative metabolism and protein metabolism. Muscle transcriptome profiles were generated from biopsy material before and after bed rest. Gene functional classification analysis was applied to a list of differentially expressed RNAs, produced by applying an uncorrected t-test to ~40,000 data points. The differentially expressed gene list was subject to a Fisher’s exact test within IPA, with the standard IPA database at that time used as the reference.

Such an input gene list is unquestionably flawed. Nevertheless, the subsequent functional classification profile appeared to be both significant and meaningful, identifying metabolism and protein pathways that are known to change in disused skeletal muscle [8]. This, in turn, allowed the authors to claim that the regulated gene list was an accurate reflection of the experiment and that more novel features were uncovered by the IPA analysis.

A second example is a study of the impact of obesity on human liver ageing [2]. Here, 657 genes were identified for which the RNA expression levels correlated with altered DNA methylation in liver samples from obese individuals. This list of genes was subject to analysis using DAVID, and a number of processes were identified, each of which would be expected if carrying out a GO analysis on any list of genes expressed in liver tissue versus the genome. Critically, the GO analysis provided central support to there being a plausible link between the samples’ ‘age score’ and important components of liver biology.

In both of these studies, a complex combination of the three aforementioned biases are at work. Most obvious is the fact that, functionally, the tissue transcriptome profiles were already biased towards the factors identified by the functional enrichment analysis. That is, they had a greater than average chance of appearing in the input (‘regulated genes’) list. Thus, the significance of the pathway analysis cannot subsequently validate, for example, the use of 40,000 unpaired t-tests.

The issue of utilizing an appropriate background comparison list is further compounded in the case of the muscle analysis [3], as it is impossible to replicate this analysis exactly because the background reference ‘gene universe’ used in IPA is frequently updated. This feature of bespoke databases makes re-evaluation of existing literature problematic. At a minimum, analyses carried out with IPA should utilize a defined background gene list that is included in the publication.

Using a free tool like DAVID, it is possible to attempt to correct for the three sources of sampling bias. This can be attempted by using a background gene list, representing the ‘universe’ of possible genes that could be called as significantly regulated in the experiment. This list should contain only factors (RNA or protein) that are both robustly ‘probed or sequenced’ (to avoid technology and detection bias) and ‘called’ as expressed (to avoid biological bias) in the experiment. After all, we do not want to carry out functional enrichment analysis of a specific tissue simply to be informed that we are studying that tissue!

Adjustments for such biases are currently imperfect. For example, a technology that could detect all transcripts with equal probability (regardless of differences in abundance) is needed to make such a background gene list. This could then be used to decide which functional categories (or pathways) are enriched over and above the bias that is already present in the tissue being studied. Currently, no technology provides a solution.

We raised this issue with the providers of ‘Ingenuity’ (a commercial web-based application for functional analysis of ‘-omics’ data) in 2011, and since then, they have allowed users to submit an experiment-specific background file (it is still important that researchers publish a record of the final list of genes that were mapped to the IPA database). Likewise, DAVID allows the user to submit a custom background file (although there is no clear guidance about its essential role). However, somewhat surprisingly, the Gene Ontology consortium website analysis tool [9] does not allow for this option, making any analysis thoroughly unreliable.

Thus, the seriousness of the bias issue remains substantially underappreciated, and guidance to reviewers and editorial boards on the importance of the three sources of sampling bias is absent. We have noted in our studies that correction tends to reduce or completely remove the significance of functional terms, and this can be an unwelcome observation after time and money has been spent on collecting and analyzing the data. Subjectively, functional enrichment analysis bias occurs very frequently in clinical genomic studies. This is typically because the sample size is limited, leading to loosening of statistical thresholds for differential expression, which is ‘compensated’ [sic] by the use of functional enrichment analysis.

It might be thought that newer technologies like RNA-seq, which is marketed as providing unbiased and global coverage, would resolve the issue of technology and detection biases. Unfortunately, RNA-seq data are neither unbiased nor global [4, 10]. Sequence-specific PCR-related challenges exist and read-depth issues mean that, for any specialized tissue, as few as 100 RNA species can dominate the raw read counts [5]. Further, because the final RNA-seq data are a statistically derived estimate of expression, any bias that impacts on the likelihood of detection will impact on the functional enrichment analysis [10].

The generation of an estimated background ‘universe’ in RNA-seq data could be achieved by removing zero-count genes, but the nature of this ‘universe’ will still depend on many factors. For example, we have noted roughly twice the number of genes detectable in adipocytes when using Affymetrix Exon ST array compared with recently published RNA-seq data from adipocytes (the latter data being presented as a comprehensive and unbiased profile of adipocyte transcripts [11]). This suggests to us that there is a lack of awareness of the limitations of RNA-seq data, and hence the validity of applying functional enrichment analysis to such data. Similarly, the application of functional enrichment analysis to ‘global’ proteomics [12], where a large part of the ‘molecular universe’ may not be detected, is fraught with problems that are currently intractable.

In conclusion, functional enrichment analysis must not be considered proof of biological plausibility or validity in the analysis of high-throughput -omics data. We strongly advocate for efforts to generate appropriate background expression ‘universes’. We also urge that background gene lists are provided for any functional enrichment analysis, and that a higher statistical threshold is used as a default, given the scale of the pre-existing biases, to avoid marginal (e.g., 1 × 10^−3^ or 1 × 10^−4^) enrichments being relied on to drive the interpretation of an experiment.

## Abbreviations

DAVID: Database for Annotation, Visualization, and Integrated Discovery
GO: Gene Ontology
IPA: Ingenuity Pathway Analysis
